# Supplementary material for: The Adsorption Behavior of Gas Molecules on Mn/N- and Mn-Doped Graphene
Source: Nanomaterials (Basel). 2024 Aug 15;14(16):1353. doi: 10.3390/nano14161353 (PMC11357153; doi:10.3390/nano14161353)
Supplement: Supplementary file 1 [file nanomaterials-14-01353-s001.zip › nanomaterials-3153033-supplementary.pdf]

The Adsorption Behavior of Gas Molecules on Mn/N-Mn-Doped Graphene

Tingyue Xie [1,2], Cuifeng Tian [1], Ping Wang [1,*], and Guozheng Zhao [2]

[1] School of Physical and Electronics Science, Shanxi Datong University, Datong 037009, China; tingyuexie@126.com (T.X.); cftian_050@sxdtdx.edu.cn (C.T.)

[2] Key Laboratory of Magnetic Molecules & Magnetic Information Materials Ministry of Education, School of Chemistry and Material Science, Shanxi Normal University, Taiyuan 030031, China; zhaoguozheng@sxnu.edu.cn

* Correspondence: wangping061226@aliyun.com

Table S1. The bond lengths (*d*, Å) between the manganese atom and the nearest adsorption site, the bond lengths (*l*, Å) of C-H or C-O before and after CH_2O adsorption on the MnSV-GP and MnN_3-GP, respectively.

Type	Site	*d* (Å)	after(*l*, Å)	initial (*l*, Å)
CH_2O/MnSV-GP				
	C-H1		1.10	1.12
	C-H2		1.10	1.12
	C-O		1.33	1.21
	O	1.86		
	C	2.03		
	C1	1.92		
	C2	1.82		
	C3	1.82		
CH_2O/MnN_3-GP				
	C-H1		1.10	
	C-H2		1.10	
	C-O		1.37	
	O	1.84		
	C	1.99		
	N1	2.02		
	N2	2.05		
	N3	2.05		

Table S2. The bond lengths (*d*, Å) between the manganese atom and the nearest adsorption site, the bond lengths (*l*, Å) of C-O before and after CO adsorption on the MnSV-GP and MnN_3-GP, respectively.

**Type**	**Site**	***d* (Å)**	**after(*l*, Å)**	**initial (*l*, Å)**
CO/MnSV-GP				
	C-O		1.16	1.14
	C	1.90		
	C1	1.83		
	C2	1.83		
	C3	1.82		
CO/MnN_3-GP				
	C-O		1.17	
	C	1.89		
	N1	1.98		
	N2	2.01		
	N3	1.98		

Table S3. The bond lengths (*d*, Å) between the manganese atom and the nearest adsorption site, the bond lengths (*l*, Å) of N-N or N-O before and after N_2O adsorption on the MnSV-GP and MnN_3-GP, respectively.

**Type**	**Site**	***d*** **(Å)**	**after(*l*, Å)**	**initial (*l*, Å)**
N_2O/MnSV-GP				
	N-N		1.19	1.14
	N-O		1.22	1.20
	N	1.88		
	C1	1.85		
	C2	1.81		
	C3	1.81		
N_2O/MnN_3-GP				
	N-N		1.26	
	N-O		1.24	
	N	1.94		
	N	1.94		
	N1	2.03		
	N2	2.02		
	N3	2.05		

Table S4. The bond lengths (*d*, Å) between the manganese atom and the nearest adsorption site, the bond lengths (*l*, Å) of S-O before and after SO_2 adsorption on the MnSV-GP and MnN_3-GP, respectively.

**Type**	**Site**	***d* (Å)**	**after(*l*, Å)**	**initial (*l*, Å)**
SO_2/MnSV-GP				
	S-O1		1.58	1.45
	S-O2		1.47	1.45
	S	2.37		
	O1	1.90		
	C1	1.81		
	C2	1.81		
	C3	1.91		
SO_2/MnN_3-GP				
	S-O1		1.55	
	S-O2		1.55	
	O1	2.07		
	O2	2.08		
	N1	2.01		
	N2	2.02		
	N3	2.03		

Table S5. The bond lengths (*d*, Å) between the manganese atom and the nearest adsorption site, the bond lengths (*l*, Å) of N-H before and after NH_3 adsorption on the MnSV-GP and MnN_3-GP, respectively.

Type	Site	*d* (Å)	after(*l*, Å)	initial (*l*, Å)
NH_3/MnSV-GP				
	N-H1		1.024	1.022
	N-H2		1.024	1.022
	N-H3		1.024	1.022
	N	2.09		
	C1	1.80		
	C2	1.80		
	C3	1.80		
NH_3/MnN_3-GP				
	N-H1		1.024	
	N-H2		1.025	
	N-H3		1.020	
	N	2.13		
	N1	1.95		
	N2	2.01		
	N3	2.01		

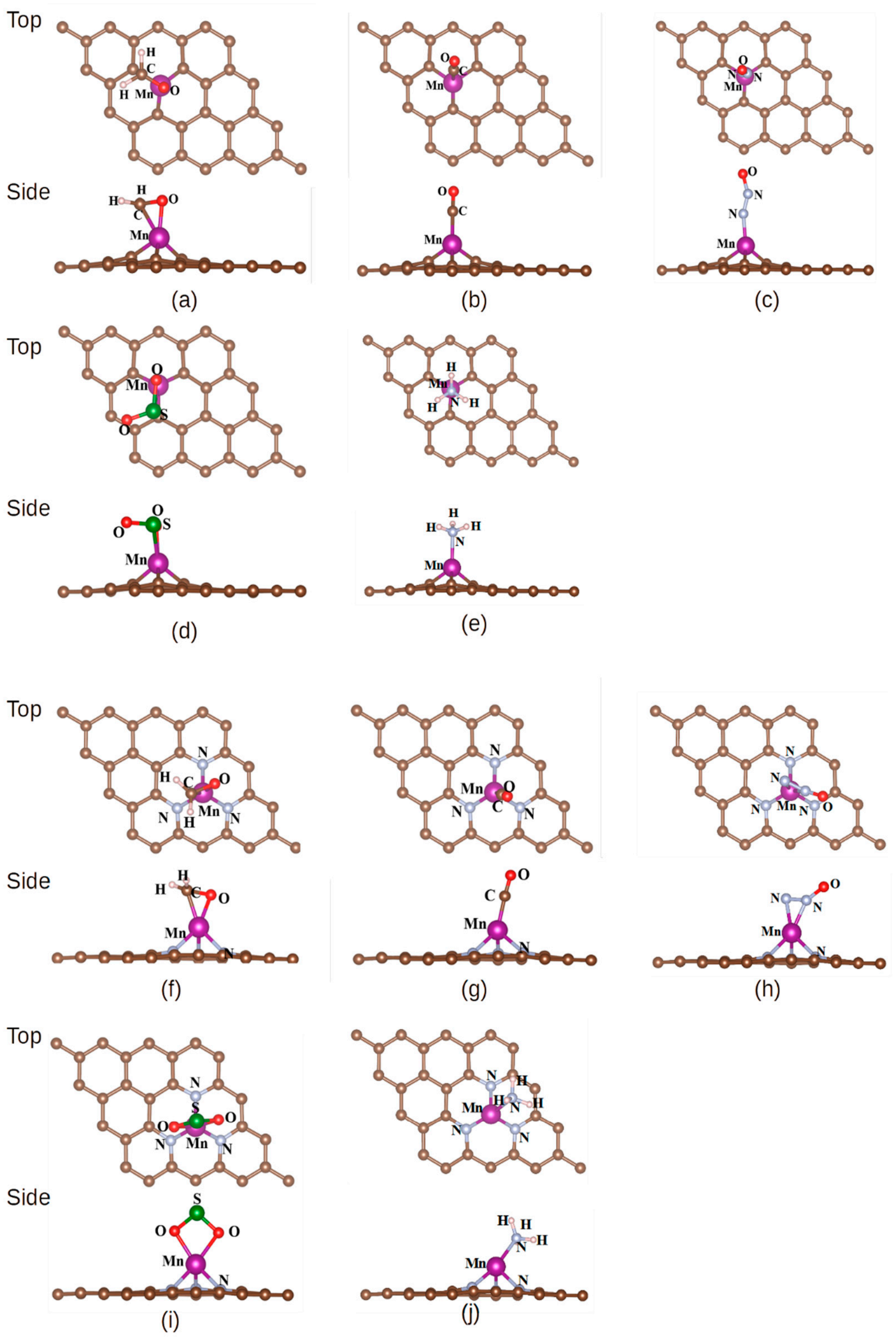

Figure S1. The optimized geometric configurations of the chemisorption system after five gas molecules adsorption on the MnSV-GP and MnN_3-GP, respectively, including (a) CH_2O, (b) CO, (c) N_2O, (d) SO_2, and (e) NH_3 on the MnSV-GP, (f) CH_2O, (g) CO, (h) N_2O, (i) SO_2, and (j) NH_3 on the MnN_3-GP, respectively.

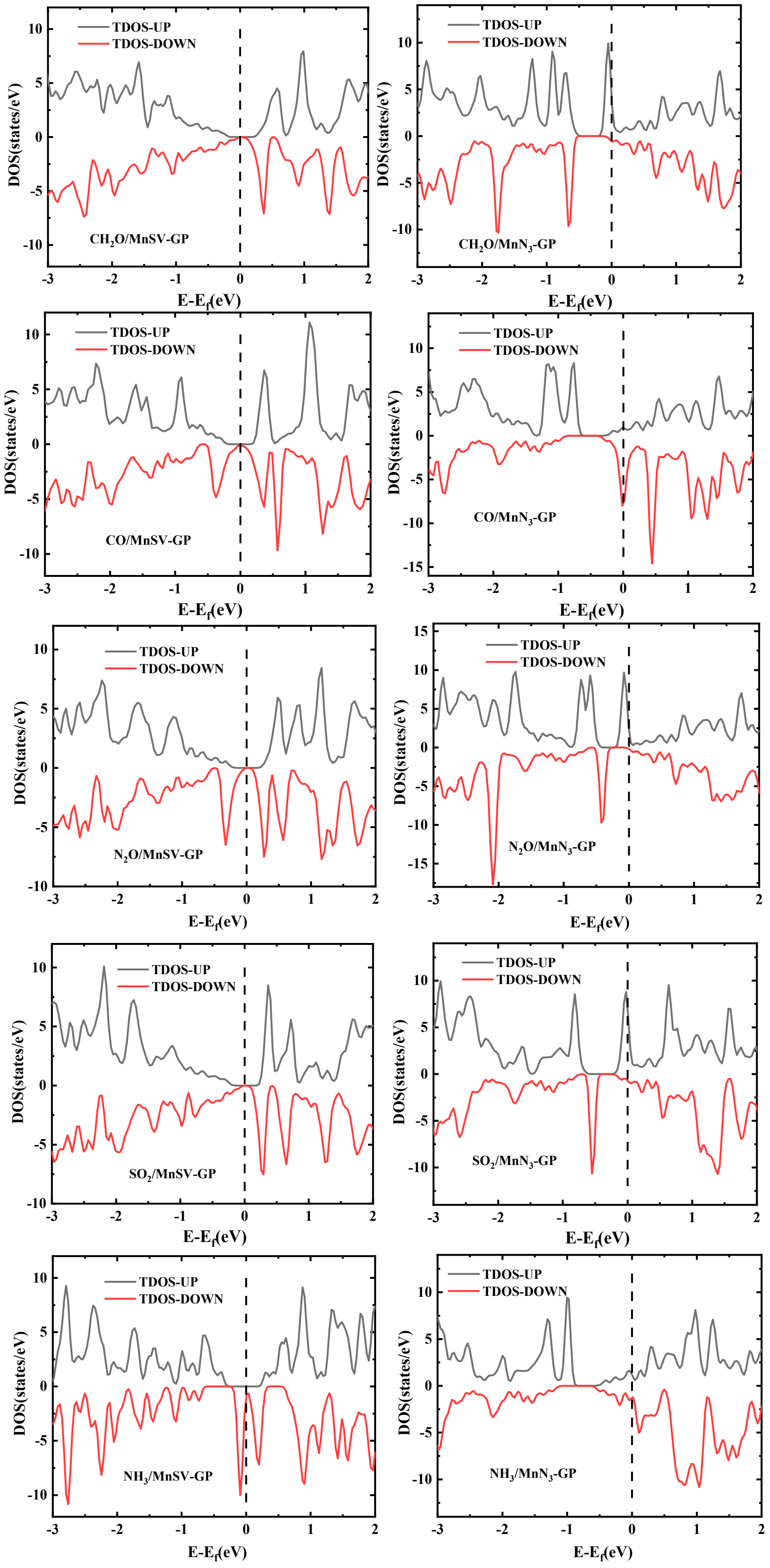

Figure S2. Total density of states of the chemisorption system for the five gases adsorption on MnSV-GP and MnN3-GP support, respectively.
